# Blood Pressure, Left Ventricular Geometry, and Systolic Function in Children Exposed to Inorganic Arsenic

**DOI:** 10.1289/ehp.1307327

**Published:** 2015-02-27

**Authors:** Citlalli Osorio-Yáñez, Julio C. Ayllon-Vergara, Laura Arreola-Mendoza, Guadalupe Aguilar-Madrid, Erika Hernández-Castellanos, Luz C. Sánchez-Peña, Luz M. Del Razo

**Affiliations:** 1 Departamento de Toxicología, Centro de Investigación y de Estudios Avanzados del Instituto Politécnico Nacional, México DF, México; 2 Hospital Español, México DF, México; 3 Departamento de Biociencias e Ingenieria CIIEMAD-IPN, México DF, México; 4 Unidad de Investigación y Salud en el Trabajo, Instituto Mexicano del Seguro Social, México DF, México

## Abstract

**Background:**

Inorganic arsenic (iAs) is a ubiquitous element present in the groundwater worldwide. Cardiovascular effects related to iAs exposure have been studied extensively in adult populations. Few epidemiological studies have been focused on iAs exposure–related cardiovascular disease in children.

**Objective:**

In this study we investigated the association between iAs exposure, blood pressure (BP), and functional and anatomical echocardiographic parameters in children.

**Methods:**

A cross-sectional study of 161 children between 3 and 8 years was conducted in Central Mexico. The total concentration of arsenic (As) species in urine (U-tAs) was determined by hydride generation–cryotrapping–atomic absorption spectrometry and lifetime iAs exposure was estimated by multiplying As concentrations measured in drinking water by the duration of water consumption in years (LAsE). BP was measured by standard protocols, and M-mode echocardiographic parameters were determined by ultrasonography.

**Results:**

U-tAs concentration and LAsE were significantly associated with diastolic (DBP) and systolic blood pressure (SBP) in multivariable linear regression models: DBP and SBP were 0.013 (95% CI: 0.002, 0.024) and 0.021 (95% CI: 0.004, 0.037) mmHg higher in association with each 1-ng/mL increase in U-tAs (*p* < 0.025), respectively. Left ventricular mass (LVM) was significantly associated with LAsE [5.5 g higher (95% CI: 0.65, 10.26) in children with LAsE > 620 compared with < 382 μg/L-year; *p* = 0.03] in an adjusted multivariable model. The systolic function parameters left ventricular ejection fraction (EF) and shortening fraction were 3.67% (95% CI: –7.14, –0.20) and 3.41% (95% CI: –6.44, –0.37) lower, respectively, in children with U-tAs > 70 ng/mL compared with < 35 ng/mL.

**Conclusion:**

Early-life exposure to iAs was significantly associated with higher BP and LVM and with lower EF in our study population of Mexican children.

**Citation:**

Osorio-Yáñez C, Ayllon-Vergara JC, Arreola-Mendoza L, Aguilar-Madrid G, Hernández-Castellanos E, Sánchez-Peña LC, Del Razo LM. 2015. Blood pressure, left ventricular geometry, and systolic function in children exposed to inorganic arsenic. Environ Health Perspect 123:629–635; http://dx.doi.org/10.1289/ehp.1307327

## Introduction

Cardiovascular disease (CVD) is a leading cause of morbidity and mortality worldwide. Inorganic arsenic (iAs) exposure has been associated with CVD in studies that have been conducted in adults (Wang et al. 2007). The occurrence of elevated, naturally occurring arsenic (As) levels in drinking water because of geothermally influenced groundwater is a cause for concern in many countries, including Argentina, Bangladesh, Chile, China, India, the United States, and México. Cardiovascular outcomes associated with drinking As-contaminated water include carotid atherosclerosis (Wang et al. 2002), cerebrovascular disease (Chiou et al. 1997), ischemic heart disease (Hsueh et al. 1998), black foot disease (Tseng 1977), and hypertension (Chen et al. 1995). Hypertension is a risk factor for CVD that also has been associated with iAs exposure. Long-term hypertension is an influence on ventricular hypertrophy development, specifically concentric hypertrophy (Verdecchia et al. 2007). Because of the left ventricular geometry pattern, concentric left ventricle hypertrophy and concentric remodeling carry the highest risk for cardiovascular events (Verdecchia et al. 2007). Hypertrophy can maintain cardiac output and left ventricular (LV) function as a compensatory mechanism, but gradually produces systolic and diastolic LV dysfunction (Artham et al. 2009). In addition to the left ventricle, the aortic root and left atrium are also modified because of chronic hypertension, and this increased left atrial mass is an independent cardiovascular risk factor (Baguet et al. 2011; Cuspidi et al. 2013). LV growth and concentric remodeling in children may have long-term consequences for cardiovascular health later in life. Recently, prehypertension was recognized to be a risk factor for hypertension and CVD development (Redwine and Falkner 2012). Until now, epidemiological evidence of anatomical and functional alterations of the heart, even in adult populations with iAs exposure, have been nonexistent. Additionally, to our knowledge, no studies have focused on CVD in children who have been environmentally exposed to iAs. Children are especially susceptible to xenobiotic toxic effects (Schwenk et al. 2003). In a mouse model, iAs exposure *in utero* accelerates CVD progression (Srivastava et al. 2007). Thus, the aim of this study was to evaluate the association of iAs exposure with BP and functional and anatomical echocardiographic parameters in children who have been exposed to iAs.

## Methods

*Child selection and recruitment*. A cross-sectional study was conducted with 192 local child residents of the Zimapan, Mexico, region. This study was approved by the regulations of the Institutional Review Board of CINVESTAV-IPN (Centro de Investigación y de Estudios Avanzados del Instituto Politécnico Nacional). Children were recruited from two different local schools during 2009; they resided in six local area towns (Calvario, Llano Norte, Tule, Dowtown, Aguacatal, and Muhi). In 2009, drinking-water iAs levels in these locations ranged from 3 to 135 μg/L. From 1993 to 2009, the historic iAs concentration in the water ranged from 3 to 398 μg/L (Armienta et al. 1997; Hernández-Zavala et al. 2008b; Valenzuela et al. 2005, 2009). In this area, high concentrations of naturally occurring iAs are frequently found in bedrock and consequently in underground and surface waters (Armienta et al. 1997). Before study enrollment, the children’s parents read and signed an informed consent form. The recruitment and interview process were carried out at schools; the participation rate (participants/eligible) in this population was ~ 95%. Only children with a minimum of 1 year of residence in the Zimapan region were eligible to participate. Children with diabetes or CVD were excluded. A fasting venous blood sample was collected. Plasma was prepared from the blood samples by centrifugation at 4°C and was stored at –80°C. The parents were interviewed by trained interviewers regarding general characteristics, with an emphasis on the source of drinking water, and detailed residential information to provide the location of each residence that they had lived in for at least 6 months, starting from 1 year before the child’s birth and ending with their current residence, the residence of the mother during pregnancy, migration, medication, and child medical history.

*Urine sample collection and arsenic speciation analysis*. First morning urine samples were collected from the participants before clinical examination, in 250-mL polypropylene containers. Urine samples were collected in each child’s home under a parent’s supervision, and were promptly transported on ice within 1–2 hr to the local health clinic where they were immediately frozen until analysis. All of the urinary As species (U-iAs^III + V^, U-MAs^III + V^, U-DMAs^III + V^) were evaluated by hydride generation (HG)–atomic absorption spectrometry (AAS) with a cryotrap (CT) for the capture and separation of hydrides (Hernández-Zavala et al. 2008a). The U-iAs, U-MAs, and U-DMAs concentrations were summed to derive the total concentration of arsenic in urine (U-tAs). As we collected first void urine, which is considered not affected by urine dilution (Saydah et al. 2013), we reported the values as nanograms As per milliliter. We used NIST (National Institute of Standards and Technology, Gaithersburg, MD, USA) Standard Reference Material (SRM) 2669 levels I and II to validate the As species analysis at low and high urine concentrations, respectively. Replicate analyses of SRM 2669 indicated a coefficient of variation < 10% and an accuracy of 95–105% for high and low standard reference values.

*Arsenic in water*. A total of 53 drinking-water samples of 53 households were collected from the six neighborhoods where children resided. Water samples were transported in ice to the CINVESTAV-IPN laboratory in Mexico City, samples were stored at –4°C and analyzed within 1 week after collection. Water samples were collected 1 month before clinical evaluations. iAs drinking-water concentrations were determined by HG–atomic fluorescence spectrometry (HG-AFS) as previously described (Le and Ma 1998). Trace elements in NIST water SRM 1643e containing 60.4 ± 0.7 μgAs/L was used for quality control.

*Lifetime arsenic exposure and* in utero *arsenic exposure*. Exposure assessment included a lifetime reconstruction of As exposure (LAsE) through a structured interview and history of drinking-water iAs concentrations at each location. LAsE is an indicator for evaluation of adverse health effects that appeared after long-term iAs exposure. iAs concentration measurements in drinking water supplies, periodically evaluated by our group since 1997, were used to estimate the average annual exposure at each location in the study area from 1997 to 2009. If no water iAs level information was available for a Zimapan location in a given year, iAs concentrations were assumed to have remained constant since the last year for which data were available. The LAsE (micrograms per liter per year) was defined as C1 × D1, where C1 is the iAs drinking-water concentration (micrograms per liter) in location 1, and D1 is the water consumption duration in years in location 1. If the children lived in two different Zimapan localities, for example, LAsE was obtained as the sum of C × D for each town, (C1 × D1) + (C2 × D2). In the case of three different Zimapan localities, calculated LAsE = (C1 × D1) + (C2 × D2) + (C3 × D3). If the children migrated outside Zimapan region, the iAs concentration was considered to be zero.

*LV geometry and function*. Clinical evaluations were performed at the local health center within 2–4 days after urine collection. Children were examined in the supine position using a cardiovascular ultrasound system. Echocardiographic studies were performed by two-dimensional guided M-mode echocardiography employing a commercially available machine (Vivid i; GE Medical Systems) according to the methods established by the American Society of Echocardiography (ASE) (Sahn et al. 1978), with appropriate transducer frequencies (1–5 MHz) for body size. Echocardiographic measurements were taken by a cardiologist who was blinded to the study design and participants’ clinical data. Measurements were obtained for aortic root diameter (ARD), left atrial diameter (LAD), LV internal diameter in diastole (LVIDD), posterior wall thickness in diastole (PWTD), and interventricular septum thickness in diastole (IVSTD). Left ventricular mass (LVM) was calculated according to the ASE-recommended formula (Sahn et al. 1978): LVM (grams): 0.8{1.04 [(LVIDD + PWTD + IVSTD)^3^ – (LVIDD)^3^]} + 0.6 g. LVM was divided by body surface area to obtain the LV mass index (LVMI). Relative wall thickness (RWT) was calculated as RWT = (2 × PWTD)/LVIDD.

LV hypertrophy (LVH) was defined as LVMI > 88.9 g/m^2^, which is above the 95th percentile reported for 192 children in the United States from 6 to 17 years old without cardiovascular disease (Daniels et al. 1995). LV geometry was categorized based on the LVMI and the RWT in combination. LVH was categorized as concentric (LVMI > 88.9 g/m^2^ and RWT ≥ 0.43) or eccentric (LVMI > 88.9 g/m^2^ and RWT < 0.43), and participants with normal geometry pattern (LVMI ≤ 88.9 g/m^2^ and RWT < 0.43) or concentric remodeling (LVMI ≤ 88.9 g/m^2^ and RWT ≥ 0.43) (Hirth et al. 2012).

Doppler echocardiography was performed for diastolic function evaluation. Early (E) and late (A) diastolic peak velocities, deceleration time (DT), and early to late diastolic peak velocity ratio (E/A) were determined for the mitral valve (MV). Early mitral annulus velocity (E´) was measured at the septal portion of the mitral annulus in an apical four chambers view using the tissue Doppler imaging technique.

*Systolic and diastolic blood pressure determination*. Systolic blood pressure (SBP) and diastolic blood pressure (DBP) were measured twice by a physician using a calibrated sphygmomanometer with an appropriately sized blood pressure cuff in the right arm in the sitting position after a 10-min rest. BP was classified using data from children in the United States identified through the master sample framework used for 1999–2000 National Health and Nutrition Examination Survey, population-based percentiles were provided by the NHBPE Program [National High Blood Pressure Education (NHBPE) Program Working Group on High Blood Pressure in Children and Adolescents 2004]. Specifically, normal SBP or DBP were defined as the respective BP < 90th percentile for age, sex, and height. Systolic or diastolic prehypertension was defined as SBP or DBP between the 90th and 95th percentile for age, sex, and height. Systolic or diastolic hypertension was defined as the respective BP ≥ 95th percentile for age, sex, and height without prehypertension or hypertension. Mixed hypertension was defined as the presence of both systolic and diastolic hypertension.

*Glucose analyses and urine and plasma nitric oxide (NO) levels*. Glucose in plasma was analyzed using an enzymatic-automated method based on spectrophotometric determination using hexokinase (Farrance 1987). Additionally, a commercial kit based on the Griess reaction (Promega Corporation) was used to determine nitrite and nitrate (NO_x_ stable metabolites) levels in urine and plasma samples. The samples were previously deproteinized and cadmium-reduced (Cortas and Wakid 1990).

*Statistical analyses*. Exploratory analyses were performed to assess data quality and consistency and the distribution of variables of interest. We tested normality of dependent variables by computing Shapiro–Wilk and Skewness/Kurtosis tests. In case of no normality, we used tools that do not require normality. The echocardiographic parameters were expressed as the geometric mean (GM) and the 95% confidence interval (CI). Other continuous variables were expressed as the GM and the range. Frequencies or percentages were used for categorical variables. We used simple linear regression models to estimate associations of BP and echocardiographic parameters with potential confounders [age, sex, body mass index (BMI) or *z*-score, fasting glucose, and NO_x_ levels] and with recent As exposure (U-tAs, U-iAs, U-MAs, and U-DMAs) or LAsE. In addition to evaluating U-tAs exposure as a continuous variable, we stratified exposure into three categories: < 35 ng/mL [where 35 ng/mL represents the Biological Exposure Index (BEI) or permissible limit for occupational As exposure (American Conference of Governmental Industrial Hygienists 2004)], 35–70 ng/mL, and > 70 ng/mL (two times the BEI value). Similarly, LAsE was categorized according to the following tertiles: < 382, 382–620, and > 620 μg/L-year. Additionally, for LVM, we assessed the association with LAsE as a continuous variable or using two categories based on 50th percentile (565 μg/L-year).

Multivariable linear regressions analyses of associations of BP or echocardiographic parameters were adjusted for covariates selected by forward method, using the Wald test with *p*-values of < 0.20; the selection was based on biological plausibility, their influence on model fit (based on *R*^2^ value), or their effect on the association between the arsenical variables and the cardiovascular parameters. This strategy combines statistical associations from the data with background knowledge about the causal network that links iAs exposure, cardiovascular outcome, and potential confounders. We included U-tAs (or U-As metabolite), and LAsE for each model. For those response variables showing alterations with U-tAs or LAsE concentrations in the first set of analyses, we further present the findings with the exposure variable stratified to further explore exposure response relationships. We adjusted for age (two categories: ≤ 5 or > 5 years), sex, and BMI (kilograms per meter squared) in all models.

No multicollinearity existed among the covariates in the model. To assess the presence of multicollinearity, we evaluated the variance inflation factor (VIF) test on all of the linear regression models. The VIF never exceeded the cut of value 5.

Analyses for validation of the multivariable regression with robust weight function were performed for all the multivariable regression models using studentized residuals to assess the heteroscedasticity of the relationship (Davidson and MacKinnon 1993). Probability values ≤ 0.05 were considered to be statistically significant, and *p* < 0.1 was considered to be marginally significant. All of the statistical analyses were performed using Stata version 10.0 (StataCorp, College Station, TX, USA).

## Results

*Study population characteristics*. Of the initial 192 participants 3 to 8 years, we excluded 1 child with an atrial septal defect, 3 children with no urine or blood samples, and 27 children with no echocardiographic determinations, leaving a final sample population of 161. It is important to note that 71% of the evaluated children were < 5 years old, and 82% were exposed to iAs *in utero.* Using standard international age- and sex-specific BMI cut points based on guidelines from the Centers for Disease Control and Prevention (2000), 65% of study participants were classified as normal weight, 8% as underweight, 18% as overweight, and 9% as obese. In total, 78% of the children had U-tAs values higher than the BEI of 35 ng/mL, and the LAsE values in this population ranged from 14 to 1,255 μg/L-year (Table 1). The echocardiographic parameters are shown in Table 2. The geometric mean for LVM was 54.6 g (95% CI: 52.8, 56.5). The mean value for ARD was 14.1 mm and for LAD 21 mm. The mean values for ejection fraction (EF) and shortening fraction (SF) were 70.7 and 39.3%, respectively.

**Table 1:** Characteristics, blood pressure, nitric oxide metabolites, total concentration of arsenic species in urine, and lifetime arsenic exposure of children in Zimapan, Mexico.

Variable	*n*	Percent or GM (range)
Sex
Male	85	53
Female	76	47
*In utero* exposure
Yes	132	82
No	29	18
Age (years)	161	5 (3–8)
≤ 5	115	71
> 5	46	29
BMI (kg/m^2^)	158	16 (12–26)
*z*-Score (percentile)	158	37 (1–99)
Blood pressure (mmHg)
Systolic BP	159	87 (70–108)
Diastolic BP	159	61 (45–80)
Hypertension classification^*a*^
Normotensive	76	48
Diastolic prehypertension	78	49
Diastolic hypertension	4	2.5
Mixed hypertension	1	0.6
Plasma glucose (mg/dL)	156	83 (64–130)
Nitric oxide metabolites
Urine NO_x_ (μM)	158	1,140 (249–4,743)
Plasma NO_x_ (μM)	156	52.0 (16.7–164)
Hemoglobin (g/dL)	157	13.8 (11.7–16.6)
Hematocrit (%)	158	39.7 (33.5–46.1)
iAs concentration in water (μg/L) (year)
2009	6 (53)^*b*^	25.9 (3–135)
1993–2009	6 (237)^*b*^	68.1 (3–313)
Urinary As ( ng/mL)
U-iAs	158	5.44 (0.57–101)
U-MAs	158	5.40 (0.21–55.7)
U-DMAs	158	46.9 (4.93–237)
U-tAs	158	59.0 (5.71–370)
U-tAs categories (ng/mL)^*c*^
< 35	34	23.4 (5.71–32.8)
35–70	68	52.7 (35.3–70.0)
> 70	56	119 (70.4–370)
U-tAs categories (ng/mL)^*d*^
< 46	54	28.6 (5.71–45.6)
46–72	52	59.8 (46.8–71.9)
> 72	52	123 (72.5–370)
Urinary As (%)
U-iAs	158	9.22 (2.33–27.2)
U-MAs	158	9.16 (1.50–18.0)
U-DMAs	158	79.5 (29.2–91.5)
LAsE (μg/L-year)	159	432 (14.0–1,255)
LAsE categories (μg/L-year)^*e*^
< 382	52	214 (14.0–382)
382–620	57	523 (391–609)
> 620	50	725 (622–1,255)
GM, geometric mean. ^***a***^Hypertension classification according to NHBPE Program criteria. ^***b***^The number of drinking-water samples taken of six localities of Zimapan area: Calvario, Llano Norte, Tule, Dowtown, Aguacatal, and Muhi; values in parentheses are total number of water samples. ^***c***^U-tAs categories based on Biological Exposure Index level. ^***d***^U-tAs categories based on tertiles. ^***e***^Lifetime arsenic exposure at the individual level based on tertiles.		

**Table 2:** Structural and functional echocardiographic parameters in children exposed to inorganic arsenic.

Variable	*n*	GM (95% CI)	Mean ± SD
Structural echocardiographic parameters
ARD (mm)	145	14.0 (13.7, 14.3)	14.1 ± 1.93
LAD (mm)	158	20.8 (20.3, 21.3)	21.0 ± 2.88
LVEDV (mL)	161	37.5 (36.0, 39.2)	38.9 ± 10.0
LVESV (mL)	161	10.5 (9.91, 11.1)	11.2 ± 3.74
LVIDD (cm)	161	3.08 (3.03, 3.14)	3.10 ± 0.34
LVISD (cm)	161	1.86 (1.82, 1.90)	1.88 ± 0.25
PWTD (cm)	161	0.74 (0.72, 0.77)	0.76 ± 0.16
IVSTD (cm)	161	0.71 (0.69, 0.72)	0.72 ± 0.11
LVM (g)	161	54.6 (52.8, 56.5)	56.0 ± 13.0
LVMI (g/m^2^)	160	67.9 (66.0, 69.8)	69.0 ± 12.3
RWT	161	0.48 (0.46, 0.50)	0.50 ± 0.15
Functional echocardiographic parameters
Systolic function
LV EF (%)	161	70.2 (68.9, 71.6)	70.7 ± 8.31
LV SF (%)	161	38.5 (37.3, 39.7)	39.3 ± 7.30
Diastolic function
Mitral E/A ratio	157	1.68 (1.62, 1.74)	1.73 ± 0.40
Mitral E DT (msec)	156	126 (122, 131)	129 ± 28.1
LV geometry pattern
Group of children [*n* (%)]
Normal^*a*^	50 (31.2)
Concentric hypertrophy^*a*^	11 (6.9)
Concentric remodeling^*a*^	99 (61.9)
Abbreviations: ARD, aortic root diameter; GM, geometric mean; IVSTD, interventricular septum thickness in diastole; LAD, left atrium diameter; LVEDV, left ventricular end diastolic volume; LVEF, left ventricular ejection fraction; LVESV, left ventricular end systolic volume; LVIDD, left ventricular internal diastolic diameter; LVISD, left ventricular internal systolic diameter; LVM, left ventricular mass; LVSF, left ventricular shortening fraction; mitral E DT, mitral E deceleration time; PWTD, posterior wall thickness in diastole; RWT, relative wall thickness. ^***a***^According to Daniels et al. (1995) and Hirth et al. (2012).			

There were no important differences between boys and girls in the parameters studied, except for the following: plasma glucose (86.7 vs. 79.9 mg/dL; *p* < 0.0001), LVM (58.63 vs. 53.07 g; *p* = 0.006), ARD (14.6 vs. 13.6 mm; *p* = 0.001), LVIDD (3.16 vs. 3.04 cm; *p* = 0.027), and the end diastolic LV volume (LVEDV) (39.2 vs. 35.8 mL; *p* = 0.030), which were all higher in boys.

Only 22% of the population reported any seafood consumption, and only 16% were exposed to secondhand smoke. Neither seafood consumption nor secondhand smoke were significant predictors of echocardiographic parameters or BP, and these variables were not included in the multivariable regression models (data not shown).

*Predictors of increased SBP or DBP*. Mean SBP and DBP were not significantly different between boys and girls (*p* > 0.1). Compared with those of normal weight, SBP was significantly higher in overweight and obese children (89 vs. 85 and 94 vs. 85 mmHg, respectively; *p* < 0.01), and DBP was higher in obese children (69 vs. 60 mmHg; *p* = 0.002). SBP and DBP both increased with age (*p* < 0.001). In accordance with NHBPE Program criteria, 48% of the children were normotensive, 49% had diastolic prehypertension, and 2.5% had diastolic hypertension. None of the children presented with systolic prehypertension or hypertension, and only one child had mixed hypertension (Table 1).

Based on a linear multivariable regression model adjusted for age (> 5 or ≤ 5 years), sex, and BMI (continuous), and urine NO_x_ (as significant predictor), SBP was positively and significantly associated with U-tAs (0.021 mmHg increase per 1 ng/mL; 95% CI: 0.004, 0.037; *p* = 0.015) but not with LAsE (Table 3). Based on a multivariable linear regression model adjusted for age, sex, BMI, and plasma glucose (a significant predictor), DBP was positively associated with U-tAs (0.013-mmHg increase per 1 ng/mL; *p* = 0.023 (Table 3).

**Table 3:** Multivariate regression analyses of systolic and diastolic blood pressure in association with chronic exposure (LAsE) and current exposure (U-tAs) to inorganic arsenic in children.

Explanatory variable	β (95% CI)	*p*-Value
Systolic blood pressure^*a*^
LAsE (μg/L-year)	–0.001 (–0.005, 0.003)	0.700
U-tAs (ng/mL)	0.021 (0.004, 0.037)	0.015
Diastolic blood pressure^*b*^
LAsE (μg/L-year)	0.0006 (–0.003, 0.004)	0.741
U-tAs (ng/mL)	0.013 (0.002, 0.024)	0.023
Abbreviations: LAsE, estimate lifetime arsenic exposure at the individual level; U-tAs, urine arsenic concentration measured concurrently with the outcomes. ^***a***^Linear regression model adjusted for age (< 5 or ≥ 5 years), sex, BMI (continuous), and urine NO_x_ (continuous); *n* = 152, *p *< 0.0001, *R*^2^ = 0.31. ^***b***^Linear regression model adjusted for age (< 5 or ≥ 5 years), sex, BMI (continuous), and plasma glucose (continuous); *n* = 147, *p *= 0.0003, *R*^2^ = 0.30.		

**Figure 1:**
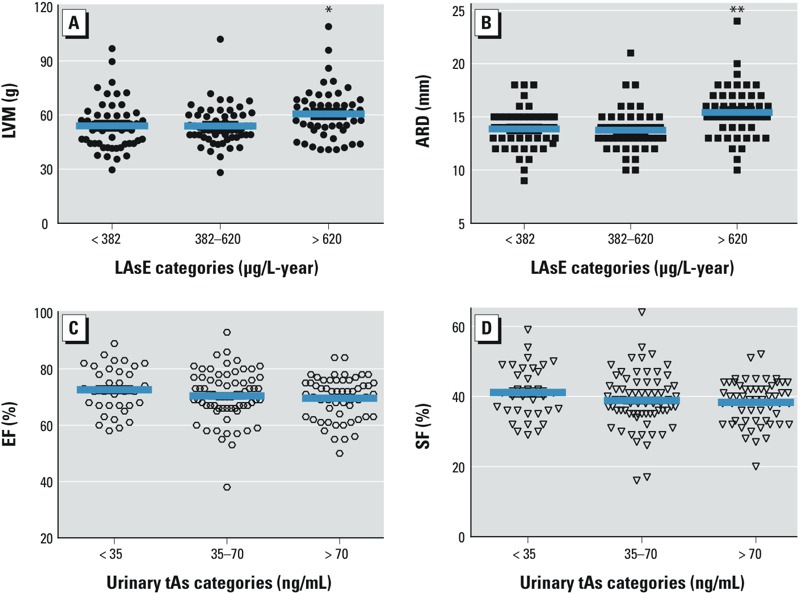
Simple linear regression analyses of left ventricular mass (*A*) and aortic root diameter (*B*) in association with lifetime arsenic exposure (LAsE) > 620 μg/L-year compared with LAsE < 382 μg/L-year (*p < 0.05, **p < 0.01). Simple linear regression analyses of ejection fraction (*C*) and shortening fraction (*D*) in association with U-tAs > 70 ng/mL compared with U-tAs < 35 ng/mL (*p* ≤ 0.1).

*LV geometric patterns and LVM*. Fifty children (31.2%) presented a normal geometric pattern, 11 (6.9%) presented concentric hypertrophy, and 99 children (61.9%) presented concentric remodeling. LVM increased with age as continuous variable (*p* < 0.001) and was greater in boys than girls (57 vs. 52 g; *p* = 0.006), and in overweight (57 vs. 54 g; *p* = 0.099) and obese (69 vs. 54 g; *p* = 0.001) groups compared with normal-weight groups. LVM (grams) increased with SBP and DBP (*p* < 0.01). In simple linear regression analyses, LVM was significant and positively related to LAsE category > 620 μg/L-year (6.53 g; 95% CI: 1.54, 11.51; *p* = 0.011) compared with LAsE < 382 μg/L-year (Figure 1A). In a multivariable linear regression model adjusted for sex, BMI, and age, LVM was higher in association with LAsE > 620 μg/L-year (5.5 g; 95% CI: 0.65, 10.3; *p* = 0.026) compared with LAsE < 382 μg/L-year (Table 4). LVM also was significantly associated with LAsE modeled as a continuous variable (0.011 g; 95% CI: 0.003, 0.020 per μg/L-year; *p* = 0.008). U-tAs was not associated with LVM (Table 4).

**Table 4:** Robust multivariable regression analyses of cardiac morphological variables or left ventricular systolic function in association with chronic exposure (LAsE) and current exposure (U-tAs) to inorganic arsenic in children.

Explanatory variable	β (95% CI)	*p*-Value
Left ventricular mass^*a*^ (g)
LAsE (382–620 μg/L-year)	0.71 (–3.44, 4.86)	0.736
LAsE (> 620 μg/L-year)	5.5 (0.65, 10.3)	0.026
U-tAs (ng/mL)	0.004 (–0.022, 0.03)	0.752
Ejection fraction^*b*^ (%)
LAsE (μg/L-year)	0.002 (–0.005, 0.008)	0.572
U-tAs (35–70 ng/mL)	–2.37 (–5.90, 1.16)	0.186
U-tAs (> 70 ng/mL)	–3.67 (–7.14, –0.20)	0.038
Shortening fraction^*c*^ (%)
LAsE (μg/L-year)	0.002 (–0.003, 0.008)	0.446
U-tAs (35–70 ng/mL)	–2.55 (–5.76, 0.65)	0.118
U-tAs (> 70 ng/mL)	–3.41 (–6.44, –0.37)	0.028
Left atrium diameter^*d*^ (mm)
LAsE (μg/L-year)	0.0013 (–0.0007, 0.0032)	0.202
U-tAs (35–70 ng/mL)	1.13 (–0.11, 2.40)	0.074
U-tAs (> 70 ng/mL)	0.46 (–0.88, 1.80)	0.499
Aortic root diameter^*e*^ (mm)
LAsE (382–620 μg/L-year)	–0.021 (–0.64, 0.60)	0.947
LAsE (> 620 μg/L-year)	1.05 (0.31, 1.80)	0.006
U-MAs (ng/mL)	0.04 (0.005, 0.070)	0.023
Abbreviations: LAsE, estimated lifetime arsenic exposure at the individual level; U-tAs, urine arsenic concentration measured concurrently with the outcomes. ^***a***^Linear regression model adjusted for age (≤ 5 or > 5 years), sex, BMI (continuous); *n* = 153, *p *< 0.0001, *R*^2^ = 0.314. ^***b***^Linear regression model adjusted for age (≤ 5 or > 5 years), sex, BMI (continuous), and SBP (continuous); *n* = 152, *p *= 0.104, *R*^2^ = 0.059. ^***c***^Linear regression model adjusted for age (≤ 5 or > 5 years), sex, BMI (continuous), and SBP (continuous); *n* = 152, *p *= 0.07, *R*^2^ = 0.07. ^***d***^Linear regression model adjusted for age (≤ 5 or > 5 years), sex, BMI (continuous), and NO_x_ in serum; *n* = 148, *p *= 0.01, *R*^2^ = 0.15. ^***e***^Linear regression model adjusted for age (≤ 5 or > 5 years), sex, BMI (continuous); *n* = 138, *p *< 0.0001, *R*^2^ = 0.265.		

*Systolic function parameters*. EF and SF were used as global systolic function parameters. EF was abnormal (≤ 50%) in only two children and SF was abnormal (< 25%) in three children. EF and SF were not associated with age, sex, or DBP in crude models. Children who were underweight had higher EF and SF values than normal weight children (*p* = 0.022 and 0.026, respectively). Compared with U-tAs < 35 ng/mL, EF (–3.0%; 95% CI: –6.56, 0.58; *p* = 0.10) and SF (–2.8%; 95% CI: –5.92, 0.33; *p* = 0.08) were inversely and marginally related to U-tAs > 70 ng/mL in crude models (Figure 1C,D). In multivariable regression analyses adjusted for SBP as well as BMI, age, and sex, EF and SF were lower in association with U-tAs > 70 ng/mL (–3.67%; 95% CI: –7.14, –0.20 and –3.41%; 95% CI: –6.44, –0.37, respectively), compared with U-tAs < 35 ng/mL.

*Left atrium and aortic root diameter*. In simple linear regression analyses LAD was significantly associated with U-tAs, 35–70 ng/mL (1.23 mm; 95% CI: 0.051, 2.40; *p* = 0.041) compared with U-tAs < 35 ng/mL. In multivariable regression analyses adjusted by age, BMI, sex, and NO_x_ in plasma, U-tAs 35–70 ng/mL was marginally associated with LAD increase (1.13 mm; 95% CI: –0.11, 2.40; *p* = 0.074) compared with U-tAs < 35 ng/mL (Table 4).

In crude linear regression analyses, ARD was positively associated with LAsE > 620 μg/L-year (1.21 mm; 95% CI: 0.45, 1.97; *p* = 0.002) compared with LAsE < 320 μg/L-year (Figure 1B). Moreover, in multivariate linear regression analyses, ARD was also associated with LAsE > 620 μg/L-year (1.05 mm; 95% CI: 0.31, 1.80; *p* = 0.006) compared with LAsE < 320 μg/L-year. ARD was significantly associated with U-MAs metabolite (0.04 mm; 95% CI: –0.005, 0.070; *p* = 0.023) but nonsignificantly associated with U-tAs (0.005 mm; 95% CI: –0.002, 0.001; *p* = 0.14) (Table 4).

## Discussion

*Arsenic exposure and BP increase*. Many prospective cohort studies have established a strong, graded, and independent positive association of BP levels with CVD risk, stroke, and premature death (Chobanian et al. 2003; Lewington et al. 2002). A study conducted in adults reported that the prevalence of hypertension was significantly associated with iAs concentration in drinking water and LAsE in endemic areas with high iAs levels in Taiwan (Chen et al. 1995). In a prospective study, maternal urinary arsenic during pregnancy was positively associated with BP in children at 4.5 years of age (Hawkesworth et al. 2013), where mean SBP was 3.69 mmHg higher (95% CI: 0.74, 6.63; *p* = 0.01) and mean DBP was 2.91 mmHg higher DBP (95% CI: 0.41, 5.42; *p* = 0.02) with each 1-μg/mL increase in maternal urine As during pregnancy. In our study, mean SBP was 21 mmHg higher (95% CI: 4, 37; *p* = 0.015) and DBP was 13 mmHg higher (95% CI: 2, 24, *p* = 0.023) with each 1-μg/mL increase in concurrently measured U-tAs in children 3–8 years of age. Interestingly, 99 children (61.9%) presented concentric remodeling and 49% presented diastolic prehypertension. The concentric remodeling prevalence in our study was higher compared with obese normotensive children (42%) at 12 years of age (Dhuper et al. 2011). However, in our study neither BP nor U-tAs was higher in concentric remodeling group compared with normal geometric pattern (data not shown). Prehypertension is associated with an approximately 3-fold greater likelihood of developing hypertension and roughly twice the number of cardiovascular events compared with normal BP (Gupta et al. 2012). It remains to be determined whether iAs exposure can produce diastolic prehypertension and/or concentric remodeling in human populations.

We did not find any statistically significant associations between BP and iAs metabolites (data not shown). Contrary to our results, an increasing risk of hypertension was reported in the highest tertile (odds ratio = 1.69; 95% CI: 1.03, 2.78) compared with the lowest tertile of U-MAs concentration (> 37.9 vs. < 11.3 μg/g creatinine) in adults in China exposed to 0–0.65 mg As/L through drinking water (Li et al. 2013). The different results between both studies could be related to the study design, different extent of exposure, uncontrolled bias in either one or both studies, and/or different iAs methylation capacities between children and adults (Sun et al. 2007).

*Arsenic exposure and LVM*. Target organ complication of hypertension reflects the degree of chronic BP elevation. LV adaption to arterial hypertension results in LV geometry responses such as concentric remodeling or concentric hypertrophy. The concentric remodeling has been associated with a poor prognosis compared with individuals who have normal LV geometry, and it is independently related to adverse cardiovascular events (Verdecchia et al. 2007). To the best of our knowledge, this is the first epidemiological study to report associations of iAs exposure with BP and LVM in children as young as 3–8 years with a high prevalence (61.9%) of LV concentric remodeling. Electrocardiographic findings indicative of cardiac repolarization abnormality have been reported in adults exposed to iAs through drinking water. A significant dose–response association between iAs exposure groups (≤ 21, 100–300, and 430–690 μg/L) and prevalence of QT prolongation (3.9, 11.1, and 20.6%, respectively), was reported in 313 adults residents of China (Mumford et al. 2007).

Our study was not designed to know the contribution of prenatal iAs exposure on cardiovascular outcomes in children. However, we found a significant association between iAs exposure and echocardiography measures of cardiac size and mass in a high percentage of children exposed prenatally (132 children; 82%). iAs exposure during prenatal and early life may have serious long-term health implications and could represent critical windows of exposure. A cohort of individuals from Antofagasta, Chile, exposed to arsenic during early life (~ 800 μg/L in drinking water) was later found to have significantly higher cardiovascular mortality than did a nonexposed control group (Yuan et al. 2007). In a study of children in Bangladesh, *in utero* and early-life exposure to arsenic through drinking water was significantly associated with the prevalence of several respiratory outcomes in the previous 12 months (Smith et al. 2013). In an experimental model, chronic exposure of mice to 100 ppb sodium arsenite for 22 weeks increased both SBP and DBP, and 43% of LVM displayed a concentric hypertrophy pattern (Sanchez-Soria et al. 2012).

*Arsenic exposure and systolic function parameters*. Although ventricular remodeling can initially be a compensatory process, it eventually leads to progressive ventricular dysfunction and heart failure (Artham et al. 2009).

In our study population, with apparently normal EF values (EF > 50%), U-tAs was significantly and inversely associated with EF and SF. In human adult populations, a significant decrease in EF was reported in occupational lead-exposed subjects compared with unexposed subjects (66.74 vs. 64.67%; *p* < 0.01; Poręba et al. 2013).

*Arsenic exposure and left atrium diameter*. Increased muscle fiber mass serves as a compensatory mechanism to help maintain contractile force and counteract elevated ventricular wall stress. However, because of increased hypertrophied wall stiffness, these benefits come at the expense of an elevated diastolic ventricular pressure, which is transmitted to the left atrium (Lilly 2011). In our study population, we found a marginal association between LAD and U-tAs 35–70 ng/mL (1.13 mm; 95% CI: –0.11, 2.40; *p* = 0.074) compared with U-tAs < 35 ng/mL. Further studies should focus on elucidating whether the LAD modification is a consequence of LVM and BP in iAs-exposed populations. The LAD increase in children may be a consequence of increased LVM and BP, which are both related to iAs exposure. In accordance with our results, LAD and LVM increase have been reported for lead-exposed workers (Poręba et al. 2013). The mechanism by which iAs increased LAD could be intimately related to the mechanism by which iAs induces hypertension.

*Arsenic exposure and ARD increase*. The ARD has been proposed to be a subclinical LV dysfunction parameter because of its association with the peak velocity of early diastolic transmitral flow (E), E/A ratio, and peak early diastolic mitral annular velocity (E´) (Masugata et al. 2011). These parameters were not significantly associated with U-tAs or LAsE in our study population (data not shown). In contrast, ARD was significantly associated with LAsE and the U-MAs urinary metabolite. U-MAs (Wu et al. 2006) and ARD (Jiang et al. 2009) have been associated with atherosclerosis in adults. Notably, previous observations in children of this study have suggested a significant relationship between U-tAs and increased carotid intima media thickness (Osorio-Yáñez et al. 2013).

*Limitations and strengths*. We cannot rule out the possibility of bias due to confounding factors such as socioeconomic status; a family history of stroke, CVD, or diabetes; or other factors. The temporal relations between exposure and the outcomes that we evaluated cannot be established because of the cross-sectional nature of our analysis. On the other hand, this is the first epidemiologic study focused on anatomic and functional cardiac parameters associated with iAs exposure in children as young as 3–8 years.

## Conclusions

Calculated LAsE and U-tAs were associated with several outcomes in our study population, specifically, BP, LVM, LAD, and ARD, and inversely associated with the systolic function parameters SF and EF. However, additional studies are warranted given our findings of an association of iAs exposure with BP and echocardiographic parameters in children. Additional research should focus on the contribution of *in utero* exposure to iAs on the cardiovascular effects.
